# Effects of organic extracts and their different fractions of five Bangladeshi plants on *in vitro* thrombolysis

**DOI:** 10.1186/s12906-015-0643-2

**Published:** 2015-04-23

**Authors:** Talha Bin Emran, Md Atiar Rahman, Mir Muhammad Nasir Uddin, Md Mominur Rahman, Md Zia Uddin, Raju Dash, Chadny Layzu

**Affiliations:** Department of Pharmacy, BGC Trust University Bangladesh, Chittagong, 4000 Bangladesh; Department of Biochemistry and Molecular Biology, University of Chittagong, Chittagong, 4331 Bangladesh; Department of Pharmacy, University of Chittagong, Chittagong, 4331 Bangladesh; Department of Pharmacy, International Islamic University Chittagong, Chittagong, 4203 Bangladesh

**Keywords:** Thrombolysis, *Trema orientalis* L, *Capsicum frutescens* L, *Urena sinuata* L. Streptokinase, Fractionation

## Abstract

**Background:**

The increasingly high incidence of ischemic stroke caused by thrombosis of the arterial vessels is one of the major factors that threaten people’s health and lives in the world. The present treatments for thrombosis are still unsatisfactory. Herbal preparations have been used since ancient times for the treatment of several diseases. The aim of this study was to investigate whether herbal preparations possess thrombolytic activity or not.

**Methods:**

An *in vitro* thrombolytic model was used to check the clot lysis effect of the crude extracts and fractions of five Bangladeshi plant viz., *Trema orientalis* L., *Bacopa monnieri* L., *Capsicum frutescens* L., *Brassica oleracea* L. and *Urena sinuata* L. using streptokinase as a positive control and water as a negative control. Briefly, venous blood drawn from twenty healthy volunteers was allowed to form clots which were weighed and treated with the test plant materials to disrupt the clots. Weight of clot after and before treatment provided a percentage of clot lysis.

**Results:**

Using an *in vitro* thrombolytic model, different fractions of five Bangladeshi medicinal plants namely *T. orientalis*, *B. monnieri*, *C. frutescens*, *B. oleracea* and *U. sinuata* showed various range of clot lysis activity. Chloroform fractions of *T. orientalis*, *B. monnieri*, *C. frutescens*, *B. oleracea* and *U. sinuata* showed highest significant (P < 0.05 and P < 0.001) clot lysis activity viz., 46.44 ± 2.44%, 48.39 ± 10.12%, 36.87 ± 1.27%, 30.24 ± 0.95% and 47.89 ± 6.83% respectively compared with positive control standard streptokinase (80.77 ± 1.12%) and negative control sterile distilled water (5.69 ± 3.09%). Other fractions showed moderate to low clot lysis activity. Order of clot lysis activity was found to be: Streptokinase > Chloroform fractions > Methanol (crude) extract > Hydro-methanol fractions > Ethyl acetate fractions > n-hexane fractions > Water.

**Conclusions:**

Our study suggests that thrombolytic activity of *T. orientalis*, *B. monnieri* and *U. sinuata* could be considered as very promising and beneficial for the Bangladeshi traditional medicine. Lower effects of other extracts might suggest the lack of bio-active components and/or insufficient quantities in the extract. *In vivo* clot dissolving property and active component(s) of *T. orientalis* and *B. monnieri* for clot lysis could lead the plants for their therapeutic uses. However, further work will establish whether or not, chloroform soluble phytochemicals from these plants could be incorporated as a thrombolytic agent for the improvement of the patients suffering from atherothrombotic diseases.

## Background

Thrombosis, the blockage of blood vessels with clots, can lead to acute myocardial infarction and ischemic stroke, the leading causes of death. Other than surgical interventions to remove or by pass the blockage, or the generation of collateral vessels to provide a new blood supply, the only treatment available is the administration of thrombolytic agents to dissolve the blood clot [1]. Thrombolytic agents that comprise tissue plasminogen activator (t-PA), urokinase (UK), streptokinase (SK) etc. are used all over the world for the treatment of these diseases. As compared to other thrombolytic drugs in India, Bangladesh and other developing countries, SK and UK are extensively used due to lower price [2,3]. All available thrombolytic agents still have significant shortcomings, including the need for large doses to be maximally effective, limited fibrin specificity and bleeding tendency. Because of the shortcomings of the available thrombolytic drugs, attempts are underway to develop improved recombinant variants of these drugs [4,5].

Day-by-day the context, concept and methods of the uses of natural products in treatment of human have undergone remarkable changes. Such changes occurred due to the fact that natural medicine or traditional medicine made a revolutionary come-back with renewed strength and vigour to play a more significant role in the management of human health [6]. Significant efforts have been concentrating towards the discovery and development of natural products from various plant and animal sources which have anti-platelet [7,8], anti-coagulant [9,10], anti-thrombotic [11] and thrombolytic activity. Epidemiologic studies have provided evidence that foods with experimentally proved anti-thrombotic effect could reduce risk of thrombosis [12]. Some plants or plant parts showing thrombolytic activity have also been reported [13]. *Trema orientalis* (Chikan), *Bacopa monnieri* (Brahmi), *Capsicum frutescens* (Lanka-marich or Marich), *Brassica oleracea* (Cauliflower), *Urena sinuata* (Kunjia, Kungooya) are native to Bangladesh. They are used as traditional medicines for cardiac diseases and blood purification. *Trema orientalis* has recently been focused due to therapeutic activity on hypoglycemic, analgesic, anti-inflammatory, anti-plasmodial, diuretic activity, laxativity, anti-convulsant, anti-helmintic, anti-sickling, antioxidant and antibacterial activity [14]. *Bacopa monnieri* possesses neuroprotective properties, nootropic activity with therapeutic implications for patients with memory loss [15]. The fruit of *Capsicum frutescens* is a cardiovascular stimulant, Capsicum assists in lowering blood pressure and breaking down cholesterol build-up [16]. Brassica vegetables possess high levels of antioxidant metabolites associated with beneficial health effects including vitamins, carotenoids, anthocyanins, soluble sugars and phenolics [16]. The biological investigation of *Urena sinuata* has not been subjected yet.

This study aims to investigate the different organic extracts of the aforementioned five Bangladeshi medicinal plants viz., *Trema orientalis* (*T. orientalis*), *Bacopa monnieri* (*B. monnieri*), *Capsicum frutescens* (*C. frutescens*), *Brassica oleracea* (*B. oleracea*), *Urena sinuata* (*U. sinuata*) for their clot lysis (thrombolytic activity) by using *in vitro* models.

## Methods

### Plant collection and identification

Leaf of *T. orientalis* (Accession No. 30816), leaf of *B. monnieri* (Accession No. 32216), fruits of *C. frutescens* (Accession No. 33018), fruits of *B. oleracea* (Accession No. 30072) and leaf of *U. sinuata* (Accession No. 36155) were collected from different parts of Chittagong region (Chakaria Upazila, University of Chittagong campus and Hill tracts area of Chittagong, Bangladesh). The plants were identified by Bangladesh Forest Research Institute (BFRI), Chittagong-4211, Bangladesh. The sample specimens of the identified plants have been preserved in the national herbarium with the mentioned accession numbers.

### Extract preparation and solvent-solvent partitioning

Plant materials were dried and ground (Moulinex Blender CK-243, Moulinex, France) into powder (50–80 mesh, 450 g approx.) to soak in 2.5 L of methanol for 14 days at room temperature (23 ± 0.5°C). Filtrate obtained through cheesecloth and Whatman filter paper No. 1 was concentrated under reduced pressure at the temperature below 45°C using rotary evaporator (Buchi Rotavapor R-200, Germany). The solvent was completely removed by using rotary evaporator (Buchi Rotavapor R-200, Germany) and obtained *T. orientalis* 7.74 g (yield 3.87% w/w), *B. monnieri* 18 g (yield 3.60% w/w), *C. frutescens* 23 g (yield 5.50% w/w), *B. oleracea* 48 g (yield 6.40% w/w) and *U. sinuata* 16 g (yield 3.4% w/w) of dried crude extracts. All of the extracts were placed in glass Petri dishes (90 × 15 mm, Pyrex, Germany).

**Figure 1:**
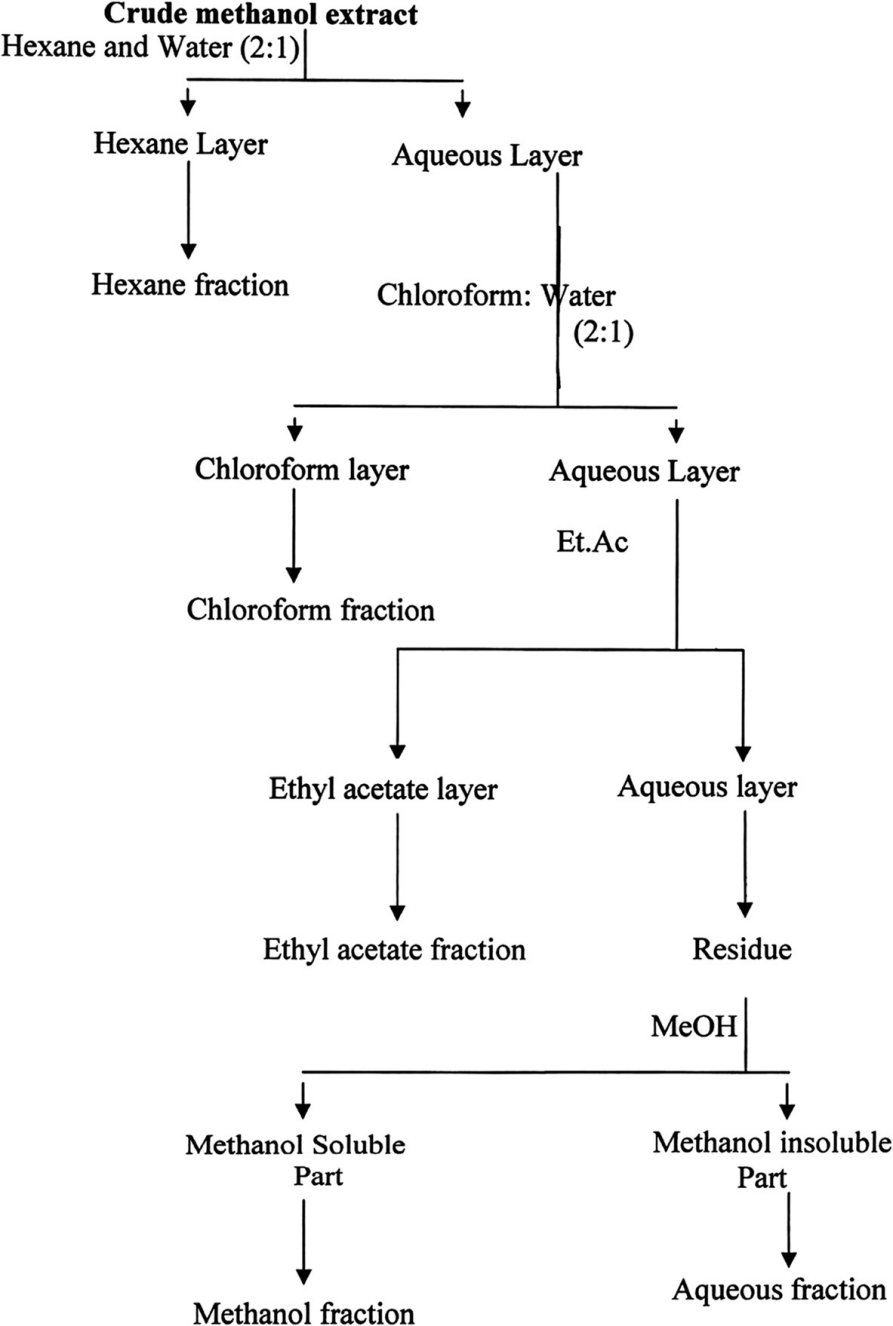
Schematic representation of the modified Kupchan partitioning of methanol crude extracts of *T. orientalis*, *B. monnieri*, *C. frutescens*, *B. oleracea* and *U. sinuata.* The crude extract was (5 g) was triturated by dissolved in 10% aqueous methanol (methanol: water; 9:1 v/v) to make the mother solution which was partitioned off successively by four solvents such as n-hexane (HX: 820 mg), chloroform (CH: 550 mg), ethyl acetate (EAT: 665 mg) and hydro-methanol (HM: 1.5 g) in order of increasing polarity by using separating funnel.

Crude extracts were undertaken for solvent-solvent partitioning by using the protocol designed by Kupchan and Tsou [17] and modified version of Wagenen et al. [17]. The crude extract (5 g) was triturated by dissolving in 10% aqueous methanol (methanol: water; 9:1 v/v) to make the mother solution which was successively partitioned by four solvents such as n-hexane, chloroform, ethyl acetate and hydro-methanol in order of increasing polarity by using separating funnel (Figure 1). Physical appearances of the fractions and their quantity after partitioning are shown in Table 1. Resulting fractions of each plant extract were dried by evaporating respective solvent using rotary evaporator. All extracts were stored at 4°C in air tight containers till further analysis [18]. A 100 mg each of the extracts was suspended in 10 mL distilled water and the suspension was shaken vigorously on a vortex mixer. The suspension was kept overnight and decanted to remove the soluble supernatant, which was filtered through a 0.22-μm syringe filter. A 100 μL of this aqueous preparation was added to the microcentrifuge tubes containing the clots to check thrombolytic activity [13].

**Table 1:** **Four different fractions of** ***T. orientalis*** **,** ***B. monnieri*** **,** ***C. frutescens*** **,** ***B. oleracea*** **and** ***U. sinuata*** **obtained after Kupchan-partitioning of the crude methanol extract**

**Plants**	**Extract/Fractions**	**Amount (g/mg)**	**Yield (% w/w)**	**Physical appearance**
***Trema orientalis***	Methanol (crude)	7.74 g	3.87	Greenish sticky mass
	Chloroform	550 mg	0.03	Red muddy structure
	n-hexane	820 mg	0.06	Deep green gummy mass
	Hydro-methanol	1.5 g	0.28	Ash like sticky mass
	Ethyl acetate	665 mg	0.05	Blackish sticky mass
***Bacopa monnieri***	Methanol (crude)	18 g	3.60	Greenish sticky mass
	Chloroform	2.25 g	0.45	Blackish sticky mass
	n-hexane	0.55 g	0.11	Deep green gummy mass
	Hydro-methanol	2.10 g	0.89	Grayish structure with presence of small needle shaped crystals
	Ethyl acetate	1.20 g	0.39	Deep red muddy structure
***Capsicum frutiescens***	Methanol (crude)	23 g	5.50	Greenish sticky mass
	Chloroform	920 mg	0.99	Green gummy mass
	n-hexane	80 mg	0.09	Blackish sticky mass
	Hydro-methanol	639 mg	0.10	Ash like sticky mass
	Ethyl acetate	90 mg	0.06	Deep green gummy mass
***Brassica oleracea***	Methanol (crude)	48 g	6.40	Greenish sticky mass
	Chloroform	1.02 g	0.68	Red muddy structure
	n-hexane	357 mg	0.11	Deep green gummy mass
	Hydro-methanol	439 mg	0.14	Blackish sticky mass
	Ethyl acetate	120 mg	0.09	Grayish structure
***Urena sinuata***	Methanol (crude)	16 g	3.4	Greenish sticky mass
	Chloroform	484 mg	0.11	Red muddy structure
	n-hexane	732 mg	0.29	Deep green gummy mass
	Hydro-methanol	846 mg	0.42	Grayish structure
	Ethyl acetate	234 mg	0.10	Blackish sticky mass

### Chemicals and reagents

To the commercially available lyophilized SK vial (Polamin Werk GmbH, Herdecke, Germany) of 15,00,000 I.U., 5 mL sterile distilled water was added and mixed properly. This suspension was used as a stock from which 100 μL (30,000 I.U.) was used for *in vitro* thrombolysis.

### Blood specimen

Whole blood (vein, 4 mL) was drawn from healthy human volunteers (*n* = 20) without a history of oral contraceptive or anticoagulant therapy using a protocol approved by the Institutional Ethics Committee of Chittagong Medical College, Chittagong-4218, Bangladesh. An earlier consent, approval number ME-CMC 2012/05, was taken from the Chittagong Medical College, Chittagong-4218, for collection of blood samples from human volunteers. Blood collection and preservation were conducted by Dr. Shafiqul Islam (Pathologist, Premium Hospital Pvt. Ltd., Chittagong). A 500 μL of blood was transferred to each of the eight previously weighed microcentrifuge tubes to form clots.

### Statement on informed consent of the donors

The volunteer donors were supplied a consent form which informed the title of the research project, name and detail contact of investigators as well as purpose of the research. Description of the research mentioning step-by-step brief of the proposed research, inclusion and exclusion criteria of the donors, whether donors will receive any therapy or not, volume of blood to be taken, possible discomfort of the puncture sites, time required for the blood sampling. Explanation was made on if future use of the research data beyond the current study is anticipated, whether this is a focus group if so the principal investigator should put a procedure in place in which the researchers caution people about the limit on confidentiality. Access to research information regarding who would have access to the collected sample, information regarding retention of sample and schedules for their disposal were also detailed. It was indicated to the consent form that the volunteers might refuse to donate blood at any time. Donor whether could withdraw his sample data was disclosed. The sample was restricted for that individual study not for future research projects was presented in the consent form. Potential harm, injuries, discomforts or inconvenience associated with donors in this study was added as informed consent statement. If there was known harm to the donors, the potential harm, current knowledge regarding the probability of the occurrence of the harm, clinical importance of the harm; and any relevant knowledge regarding the probability of reversibility; for example the possibility of bruising or swelling while giving blood, or some other discomforts at the site where blood is drawn and that there might be minimal chance of infection, and that these discomforts were brief and transient were also added. Potential benefits of the donors, not directly, but the society in general or individuals with a similar condition might benefit from the results of this study was explained. Treatment alternative and possibility of the research was described. Confidentiality statement was included in the consent form in the way that “confidentiality will be respected and no information that discloses the identity of the participant will be released or published without consent unless required by law of states. The legal obligation includes a number of circumstances, such as suspected child abuse and infectious disease, expression of suicidal ideas where research documents are ordered to be produced by a court of law and where researchers are obliged to report to the appropriate authorities. In those rare instances where it will not be possible to assure complete confidentiality”, the limits on this obligation were carefully explained. Reimbursement issue was also mentioned whether the donors or their parents may be offered money for reasonable out-of-pocket expenses for example, transportation costs, meals, etc. Finally detail contact (name, area code and phone number) of investigators was provided in case of any questions of the donors about this study. The consent form was concluded with major questions on above disclosures in Yes/NO form followed by the signature (with date) of the donor.

### Clot lysis

Experiments for clot lysis were carried as reported previously [19]. Briefly, 4 mL venous blood drawn from the healthy volunteers was distributed in eight different pre-weighed sterile microcentrifuge tubes (0.5 mL/tube) and incubated at 37°C for 45 minutes. After clot formation, serum was completely removed without disturbing the clot and each tube having clot was again weighed to determine the clot weight (clot weight = weight of clot containing tube – weight of tube alone). To each microcentrifuge tube containing pre-weighed clot, 100 μL of different organic extracts of the five plants (*T. orientalis*, *B. monnieri*, *C. frutescens*, *B. oleracea* and *U. sinuata*) were added separately. As a positive control, 100 μL of SK and as a negative non-thrombolytic control, 100 μL of distilled water were separately added to the control tubes numbered. All the tubes were then incubated at 37°C for 90 minutes and observed for clot lysis. After incubation, released fluid was removed and tubes were again weighed to observe the difference in weight after clot disruption. Difference obtained in weight taken before and after clot lysis was expressed as percentage of clot lysis. The experiment was repeated with the blood samples of the twenty (20) healthy volunteers.

### Statistical analysis

The significance between % clot lysis by SK and plant extracts was tested by the paired t-test analysis using the software SPSS, version 18.0 (SPSS for Windows, Version 18.0, IBM Corporation, New York, USA). Data are expressed as mean ± standard deviation. The mean difference between positive and negative control was considered significant at P values < 0.05 and 0.001.

## Results

**Figure 2:**
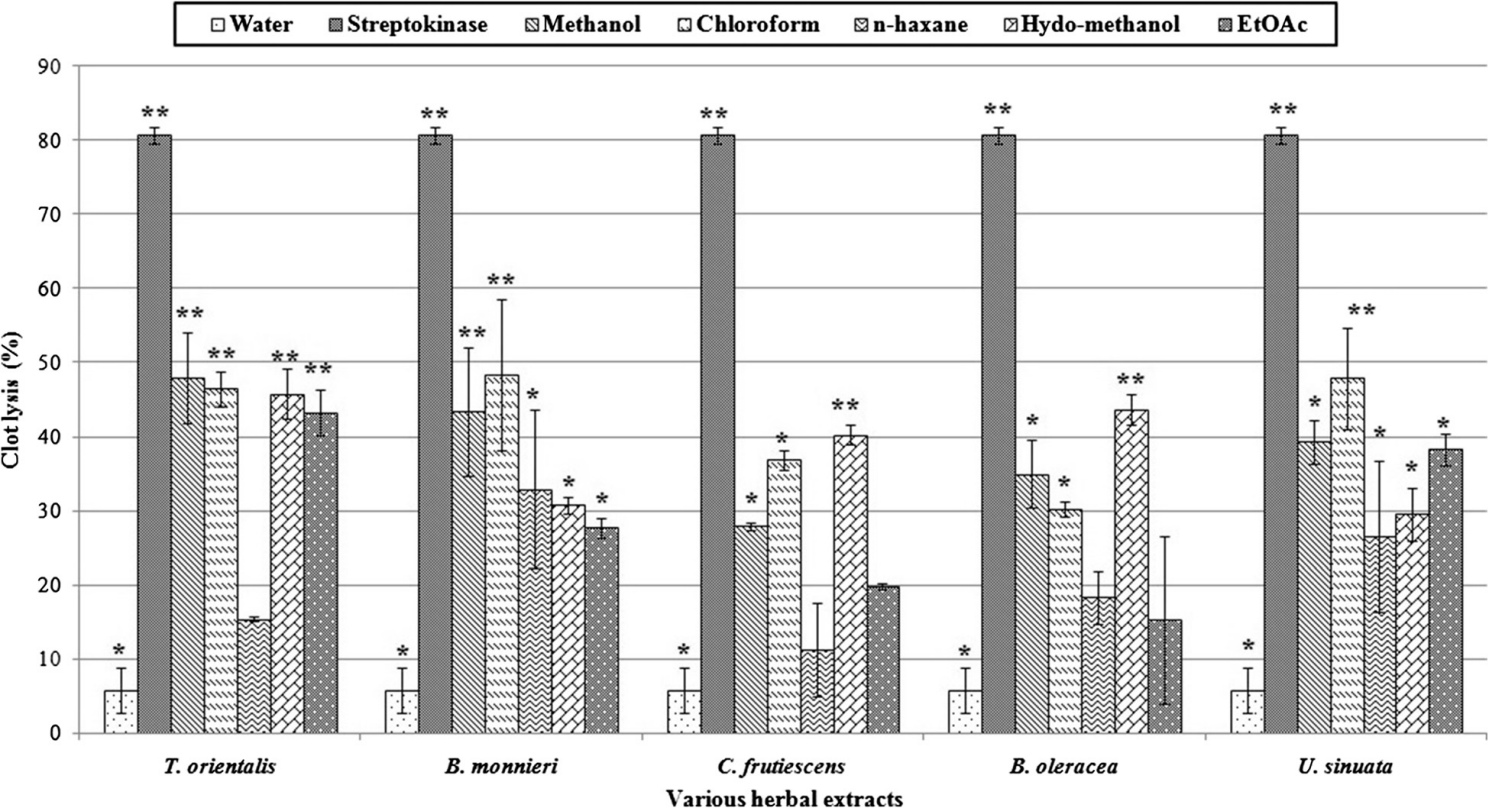
Clot lysis by streptokinase, water, various organic extracts and their different fractions. Effects of drugs on dissolution of clots prepared from blood of normal individuals. Maximum clot lysis (80.77 ± 1.12%) was observed in clot treated with streptokinase (SK). Among herbal drugs chloroform fractions of *T. orientalis*, *B. monnieri*, *C. frutescens*, *B. oleracea* and *U. sinuata* showed highest significant (P < 0.05 and P < 0.001) clot lysis activity viz., 46.44 ± 2.44%, 48.39 ± 10.12%, 36.87 ± 1.27%, 30.24 ± 0.95% and 47.89 ± 6.83% respectively. Sterile distilled water (as a negative control) showed 5.69 ± 3.09% clot lysis. Values are mean ± SD, (n = 5); *P < 0.05, **P < 0.001, Dunnet test as compared to control (positive and negative). Data were processed by paired t-test analysis by using SPSS for windows, version 18.0.

Addition of 100 μL SK (positive control) to the clots along with 90 minutes of incubation at 37°C, showed 80.77 ± 1.12% clot lysis. Sterile distilled water (negative control) treated-clots showed only 5.69 ± 3.09% clot lysis which is very negligible. The mean differences in clot lysis percentage between positive and negative control was very significant (P values < 0.001 and 0.05 respectively). Chloroform fractions of *B. monnieri* showed the highest (48.39%) significant (P values < 0.001) clot lysis activity among the other extracts. Chloroform fractions of *U. sinuata* (47.89%) and *T. orientalis* (46.44%) also gave significant (P values < 0.001) clot lysis which is almost similar to that of *B. monnieri* chloroform fractions*.* Chloroform fractions of *C. frutiescens* and *B. oleracea* have the moderate but significant (P values < 0.05) clot lysis activity and the values were respectively 36.87% and 30.24%. However, hydro-methanol fractions of *T. orientalis, C. frutiescens* and *B. oleracea* have significant clot lysis activity viz. 45.78%, 43.70%, 40.29% respectively (P values < 0.001). n-hexane fractions of only *B. monnieri* showed moderate also significant (P values < 0.05) clot lysis 32.88% whereas ethyl acetate fractions of *T. orientalis* (43.29%) and *U. sinuata* (38.29%) (P values < 0.001 and 0.05 respectively) had significant clot lysis activity. Other ethyl acetate fractions showed very lower clot lyses which were insignificant compared to negative control. Crude methanol extract of all plants except *C. frutiescens* had significant (P values < 0.001 and 0.05) clot lysis activity. Percent clot lysis obtained after treating the clots with different organic extracts and appropriate controls is shown in Table 2 and represent in Figure 2.

**Table 2:** **Effect of herbal extracts and their different fractions on** ***in vitro*** **clot lysis**

**Herb/Drug**	**Extract/Fractions**	**% Clot lysis (mean ± S.D)**	**P-values (two-tailed) when compared to negative control (water)**
**Streptokinase (+ve control)**	-	80.77 ± 1.12**	<0.001
**Water (−ve control)**	-	5.69 ± 3.09^*^	<0.05
***Trema orientalis***	Methanol (crude)	47.88 ± 6.12**	<0.001
	Chloroform	46.44 ± 2.44**	<0.001
	n-hexane	15.37 ± 0.29	
	Hydro-methanol	45.78 ± 3.32**	<0.001
	Ethyl acetate	43.29 ± 2.98**	<0.001
***Bacopa monnieri***	Methanol (crude)	43.38 ± 8.69**	<0.001
	Chloroform	**48.39 ± 10.12** **	< 0.001
	n-hexane	32.88 ± 10.72*	< 0.05
	Hydro-methanol	30.73 ± 1.13*	< 0.05
	Ethyl acetate	27.68 ± 1.34*	< 0.05
***Capsicum frutiescens***	Methanol (crude)	27.96 ± 0.50*	< 0.05
	Chloroform	36.87 ± 1.27*	< 0.05
	n-hexane	11.23 ± 1.29	
	Hydro-methanol	40.29 ± 1.26**	< 0.001
	Ethyl acetate	19.82 ± 0.49	
***Brassica oleracea***	Methanol (crude)	34.96 ± 4.60*	< 0.05
	Chloroform	30.24 ± 0.95*	< 0.05
	n-hexane	18.30 ± 3.55	
	Hydro-methanol	43.70 ± 1.97**	< 0.001
	Ethyl acetate	15.29 ± 11.29	
***Urena sinuata***	Methanol (crude)	39.30 ± 2.87*	< 0.05
	Chloroform	47.89 ± 6.83**	< 0.001
	n-hexane	26.58 ± 10.27*	< 0.05
	Hydro-methanol	29.51 ± 3.62*	< 0.05
	Ethyl acetate	38.29 ± 2.18*	< 0.05

Values are mean ± SD, (n = 5); *P < 0.05, **P < 0.001, Dunnet test as compared to control (positive and negative). Statistical representation of the effective clot lysis percentage by herbal preparations, positive thrombolytic control (Streptokinase) and negative control (sterile distilled water) processed by paired t-test analysis (Dunnet test). Bold text indicates the highest clot lytic activity of chloroform extract of *Bacopa monnieri*.

## Discussion

Numbers of pharmaceuticals approved by the Food and Drug Administration (FDA) currently have origins to plant sources. Based on the reported immunomodulatory effects, the most important role for plant-derived compounds has emerged in recent times and has led to the precise scientific examination to determine efficacy and safety [20,21]. A number of plants source especially several fruits and vegetables have been studied for their supplements having anti-coagulant, anti-platelet and fibrinolytic activity and there is evidence that consuming such food leads to prevention of coronary events and stroke [22-24]. There are several thrombolytic drugs obtained from various sources. In our present study, four different extracts of five diverse plants showed the thrombolytic activity among which the crude extracts and fractions of *T. orientalis and B. monnieri,* had the significant activity than the other plants. *B. oleracea and U. sinuata* have moderate thrombolytic activity. The maximum clot lysis activity was mostly observed in chloroform fractions that mean chloroform soluble compounds are mainly responsible for the thrombolytic activity. Hydro-methanol fractions are next to the chloroform fractions in clot lysis effect. It is evident that there are bacterial contaminants of plants which have plasminogen receptors that bind plasminogen. Cell surface bound plasminogen is easily activated to plasmin, which could lead to fibrinolysis [25] although some other plants exert their thrombolytic or fibrinolytic effects via their content of certain fibrinolytic proteases enzymes. However, bacterial plasminogen activator: staphylokinase, streptokinase, act as cofactor molecules that contribute to exosite formation and enhance the substrate presentation to the enzyme. Staphylokinase activates plasminogen to dissolve clots, also destroys the extra-cellular matrix and fibrin fibers that hold cells together [26,27]. Coincidentally, crude methanol extract of *T. orientalis* showed antibacterial activity against *Staphylococcus aureus*, *Staphylococcus epidermidis* [28]. *B. monnieri* was also found to have very strong antibacterial effect against *Staphylococcus aureus* [29]. Koffi-Nevry et al. [30] noticed very promising effect of *C. frutescens* methanol extracts against *Staphyloccus aureus*. Rahman et al. [13] has also linked the thrombolytic effects of plant materials with the anti-staphylococcal effects although some of these plants/plant products are modified further in order to use as thrombolytic drugs which are more site specific and effective [31]. Sibi et al. [32] have also determined the *in vitro* antibacterial activities of Broccoli (*B. oleracea*) against food borne bacteria. Individual chemical component-activity relationship, which can explore the other new clue for the observed thrombolytic effects of these plants, will be the next step of the research follow-up of our continuous study.

## Conclusion

We have described the thrombolytic activity of *T. orientalis*, *B. monnieri, C. frutescens*, *B. oleracea* and *U. sinuata*, which are beneficial in the Bangladeshi traditional medicine, validated in this study by *in vitro* blood clots lysis activity of their different organic fractions*.* However, *T. orientalis* and *B. monnieri* showed the promising thrombolytic effects to be studied further for therapeutic applications. Identification of their bioactive components and a dose–response relationship study in *in vivo* model is claimed to use these indigenous sources for pharmaceutical preparations. Chloroform soluble phytochemicals from these plants will be focused especially whether the fractions could account for the incorporation as a thrombolytic agent to the improvement of the patients suffering from atherothrombotic diseases.
